# Visualizing Microorganism-Mineral Interaction in the Iberian Pyrite Belt Subsurface: The *Acidovorax* Case

**DOI:** 10.3389/fmicb.2020.572104

**Published:** 2020-11-26

**Authors:** Cristina Escudero, Adolfo del Campo, Jose R. Ares, Carlos Sánchez, Jose M. Martínez, Felipe Gómez, Ricardo Amils

**Affiliations:** ^1^Centro de Biología Molecular Severo Ochoa (CBMSO, CSIC-UAM), Universidad Autónoma de Madrid, Madrid, Spain; ^2^Departamento de Planetología y Habitabilidad, Centro de Astrobiología (CAB, INTA-CSIC), Madrid, Spain; ^3^Departamento de Electrocerámica, Instituto de Cerámica y Vidrio, CSIC, Madrid, Spain; ^4^Departamento de Física de Materiales, Universidad Autónoma de Madrid, Cantoblanco, Madrid, Spain

**Keywords:** fluorescence *in situ* hybridization, confocal Raman microscopy, Raman-FISH, subsurface, *Acidovorax*, pyrite, geomicrobiology

## Abstract

Despite being considered an extreme environment, several studies have shown that life in the deep subsurface is abundant and diverse. Microorganisms inhabiting these systems live within the rock pores and, therefore, the geochemical and geohydrological characteristics of this matrix may influence the distribution of underground biodiversity. In this study, correlative fluorescence and Raman microscopy (Raman-FISH) was used to analyze the mineralogy associated with the presence of members of the genus *Acidovorax*, an iron oxidizing microorganisms, in native rock samples of the Iberian Pyrite Belt subsurface. Our results suggest a strong correlation between the presence of *Acidovorax* genus and pyrite, suggesting that the mineral might greatly influence its subsurface distribution.

## Introduction

Interest in deep continental subsurface geomicrobiology has grown in the last decades and led to an increase in the amount of information on these environments. Today, we know that life in deep continental subterranean environments is widespread, diverse and active (Kieft, 2016; Escudero et al., 2018a; Magnabosco et al., 2018). However, more in-depth analyses are still needed to better understand the functioning of subsurface ecosystems.

One of the great unknowns that needs to be studied in depth is how mineralogy affects the distribution of the microbial populations, and conversely, how a specific microbial population affects the mineralogy of the system. Some investigations have shown that deep subsurface biodiversity depends directly on the mineralogical composition of the subsurface (Jones and Bennett, 2014, 2017; Rempfert et al., 2017; Casar et al., 2020). Minerals, one of the main sources of electron donors and acceptors in these oligotrophic ecosystems, could determine which metabolisms are carried out and, therefore, which microorganisms can inhabit a certain subsurface micro niche (Jones and Bennett, 2014). Actually, hydrogen produced by water-rock interaction (among others processes) is believed to be one of the principal drivers of subsurface environments (Pedersen, 1997; Stevens and McKinley, 2000; Chapelle et al., 2002; Mayhew et al., 2013; Schrenk et al., 2013). Nonetheless, only a very limited number of studies have addressed the possible existence of a correlation between the presence of a given microorganism and hence its metabolism with the presence of a specific mineral (Jones and Bennett, 2014, 2017; Casar et al., 2020) and, to the best of our knowledge, we are unaware of any research analyzing how the microorganism-mineral interaction can affect the ecosystem.

Iberian Pyrite Belt Subsurface Life Detection (IPBSL) is a drilling project designed to characterize the Iberian Pyrite Belt (IPB) subsurface biosphere (Amils et al., 2013), whose activity is likely the origin of the peculiar characteristics of the Río Tinto basin (Fernández-Remolar et al., 2008a,b). Río Tinto is an acidic river with a high concentration of heavy metals in solution, mainly ferric iron, which is responsible for its characteristic red water and the constant pH of around 2.3 of the river (Amils et al., 2002). Operating as a natural bioreactor, the microbial community of the IPB subsurface makes use of the high concentration of metal sulfides, primarily pyrite, generating a high concentration of ferric iron in anaerobic conditions. Thus, the analysis of the microorganism-mineral interaction in IPB subsurface samples is essential to understand how this ecosystem operates.

Throughout the IPBSL project, a multimethodological approach was used to determine the biodiversity and its distribution along the column as well as to analyze the main energy sources available for life in the IPB (Amils et al., 2013). One of the most abundant microorganisms detected in the IPB subsurface was *Acidovorax* (Amils et al., under review), a circumneutral nitrate-dependent Fe^2+^ oxidizing bacteria (Straub et al., 2004; Kappler et al., 2005; Chakraborty and Picardal, 2013), which suggests that it may play an important role in the ecosystem. Hence, it would be interesting to know the *Acidovorax* distribution within the IBP subsurface and the minerals with which could be interacting.

Fluorescence *In Situ* Hybridization (FISH) techniques allow the visualization and identification of microorganisms in their natural environment (Moter and Göbel, 2000), which can be especially useful when analyzing microbial distribution in heterogeneous environments such as hard rock subsurface (Escudero et al., 2018b). However, the geochemical characterization of the sample is not possible using fluorescence microscopy. There are, nevertheless, other microscopy techniques that allow chemical analysis of the sample such as Confocal Raman Microscopy (CRM) (Dieing et al., 2011), although their usefulness is limited when identifying microorganisms. The combination of both techniques has been described previously, leading to the term Raman-FISH (Huang et al., 2007). Thanks to Raman-FISH, the metabolic characterization of microorganisms of interest was possible using isotopically labeled substrates and specific FISH probes (Huang et al., 2007). However, until now, Raman-FISH had never been applied to native rock subsurface samples.

In this study, fluorescence microscopy and Raman-FISH techniques were employed on natural rock samples from the IPB subsurface to analyze *in situ* whether mineralogy influences the distribution of members of the genus *Acidovorax* inhabiting the ecosystem.

## Materials and Methods

### Drilling and Sampling

Drilling of borehole 10 (BH10) and sampling was previously described in detail by Amils et al. (2013) and Puente-Sánchez et al. (2018). Briefly, cores were transported to the field laboratory in anaerobic conditions using plastic bags in which oxygen was displaced with N_2_. Cores were placed in an anaerobic chamber (N_2_ 95%, H_2_ 5%) decontaminated with Virkon S (Antec International Limited), ethanol and a 50:50 bleach:water solution before the introduction of each core. Then, central and untouched portions of the core were obtained using hydraulic core splitter and a rotary hammer with sterile bits, controlling the temperature (40°C maximum) with an infrared thermometer. Potential contamination of cores was monitored routinely by Ion Chromatography, used to detect sodium bromide, which was added to the drilling fluid as a contamination tracer. Additional controls carried out by DNA massive sequencing showed that no microbial contamination was introduced during sampling.

Rock samples for FISH analyses, consisting of small fragments and rock powder, were obtained from the central portion of the core with a sterile chisel. Rock samples were fixed in the field laboratory with 4% formaldehyde in Mackintosh minimal media [(NH_4_)_2_SO_4_ 132 mg/l, KH_2_PO_4_ 27 mg/l, MgCl_2_^∗^6H_2_O 53 mg/l, CaCl_2_^∗^2H_2_O 147 mg/l, pH 1.8] for 2 h at 4°C. After fixation, rock samples were washed with Mackintosh minimal media to remove the fixation agent, PBS (NaCl 8 g/l, KCl_2_ 0.2 g/l, Na_2_HPO_4_ 1.44 g/l, KH_2_PO_4_ 0.24 g/l) to neutralize the pH and, finally, stored in ethanol:PBS (1:1) at −20°C until further processing.

### Fluorescence Microscopy

Samples were sonicated (20 s, 3 cycles, one pulse per second at 20% intensity) to detach microorganisms from rock and 100 μl of supernatant was filtered in 0.22 μm black membranes (Millipore, Germany) in aseptic conditions. Filters were washed with PBS and absolute ethanol and then air dried. Catalyzed Reported Deposition-FISH (CARD-FISH) experiments and their respective controls were performed in membrane filters as previously described in Escudero et al. (2018b). ACI145 and ACI208 probes (Amann et al., 1996; Schulze et al., 1999) (Biomers, Germany) were used to target the maximum number of microorganisms of the *Acidovorax* genus since both probes complement each other. Samples from the first 300 m of BH10 were analyzed.

Biofilm detection was performed directly on fixed rock samples. Rocks were ground gently in a sterile mortar and pestle under sterile conditions to the size of grains of sand. Double labeling of oligonucleotide probes-FISH (DOPE-FISH) was carried out as described previously in Escudero et al. (2018b) with double CY3-labeled ACI145 and ACI 208 probes. Polysaccharides were visualized by Fluorescence Lectin Binding Assay (FLBA), using Concanavalin A lectin conjugated with fluorescein isothiocyanate (FITC) fluorophore (Vector Laboratories, United States). Fe^3+^ was stained with 2 μM Ferrum 430^TM^ (Ursa BioScience, United States) diluted in ethanol/H_2_O 90/10 (%Vol/Vol) for 10 min. Samples were washed with ethanol/H_2_O 90/10 (%Vol/Vol) and let air dry in darkness.

Filters and powdered rock samples were counterstained with Syto9 (Thermo Fisher Scientific, United States) as manufacturer recommended and covered with a mix of 1:4 Vectashield (Vector Laboratories, United States): Citifluor (Citifluor, United Kingdom). Filters were mounted onto glass slides and rock samples were mounted onto μ-slides 8-well glass bottom (Ibidi, Germany).

Samples were imaged with a confocal laser scanning microscope LSM710 coupled with an inverted microscope AxioObserver (Carl Zeiss, Jena, Germany) and equipped with diode (405 nm), argon (458/488/514 nm) and helium and neon (543 and 633 nm) lasers. Images were collected with 63×/1.4 oil immersion lens. Fiji software was used to project the stacks to 2D images (Schindelin et al., 2012).

### Raman-FISH

CARD-FISH was performed in powdered rocks as described above for biofilm detection. However, samples were not immobilized in agarose or covered with antifade mounting medium to avoid any interference of either agarose and Vectashield:Citifluor mixture with Raman spectroscopy. Samples were mounted onto a glass Micro-Slide Field Finder, on which a rectangular-coordinate grid pattern was drawn (EMS, United Kingdom).

Rock samples were imaged using a confocal laser scanning microscope LSM710 as described above but coupled with a vertical microscope AxioObserver (Carl Zeiss, Jena, Germany). Images were collected with a 50×/1.4 air lens.

Once the cells were located in the coordinate system by CLSM, samples were analyzed by confocal Raman microscopy after bleaching the fluorophore as described in Huang et al. (2007). Raman analyses were performed by using a confocal Raman microscope Witec alpha-300RA (Witec, Germany). Raman spectra were obtained using a 532 nm excitation laser and a 100X/0.95 air objective lens. The incident laser power was 2 mW and the acquisition time for a single Raman spectrum was 3 s (1 pixel, 1 μm^2^). A total of 23 Raman mappings were carried out in the different samples examined. Collected spectra were analyzed by using Witec Control Plus software (Witec, Germany). Comparison of Raman band intensities and Raman shifts between different Raman spectra were analyzed using Lorentzian peak curves to fit the Raman bands and the results represented by derived Raman image. In each Raman mapping, spectra were divided in two groups: *Acidovorax*+, which includes those spectra that present *Acidovorax* signal; and *Acidovorax*-, which includes those spectra that do not present *Acidovorax* signal. From each group, the average of the Ag/Eg values of the pyrite Raman bands as well as the average of the Ag and Eg Raman peaks shift values were calculated. As a result, paired data from each Raman mapping were obtained and represented in box plots. Additionally, Pearson’s correlation coefficient (PCC) was calculated from Raman images by using the ImageJ software plugins JACoP (Just Another Colocalization Plugin) (Bolte and Cordeliers, 2006) and EzColocalization (Stauffer et al., 2018). ImageJ was also used to generate the 2D images (z-projections) from Raman image stacks.

## Results

### *Acidovorax* Distribution Along BH10

**FIGURE 1:**
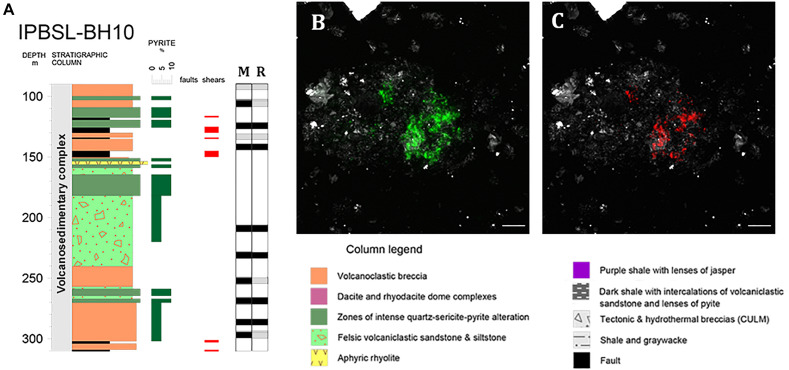
*Acidovorax* genus in the IPB subsurface analyzed by CARD-FISH. **(A)** *Acidovorax* distribution along BH10 column. Black and gray squares indicate presence or absence of microorganisms at a determined depth respectively in membrane filters (M) and rocks (R). **(B,C)** *Acidovorax* sp. at 206.6 mbs. In green, Syto9 stain. In red, CARD-FISH signal. In gray, reflection. Scale bars, 5 μm.

CARD-FISH analysis was performed to determine the depths at which *Acidovorax* genus is present throughout the first 300 meters below surface (mbl) of the IPB column (Figure 1). These initial CARD-FISH experiments were performed on membrane filters in which the supernatant of the sonicated rock samples was filtered to detect the detached microorganisms from the rock samples. Members of the *Acidovorax* genus were detected at 10 of 14 depths analyzed (Figure 1A).

### Raman-FISH on IPB Subsurface Samples

**FIGURE 2:**
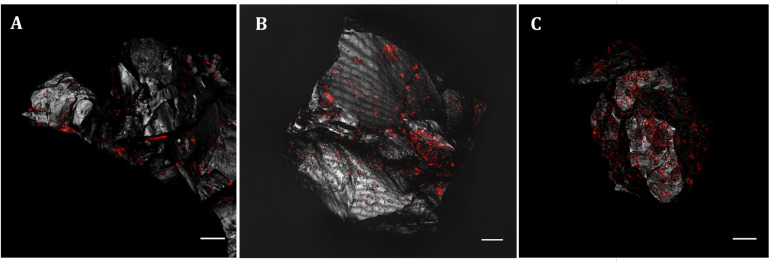
*Acidovorax* genus members detected by CARD-FISH in IPB subsurface rock samples at –139.4 m **(A)**, –206.6 m **(B)** and –284 m **(C)**. In red, CARD-FISH signal. In gray, reflection. Scale bars, 10 μm.

**FIGURE 3:**
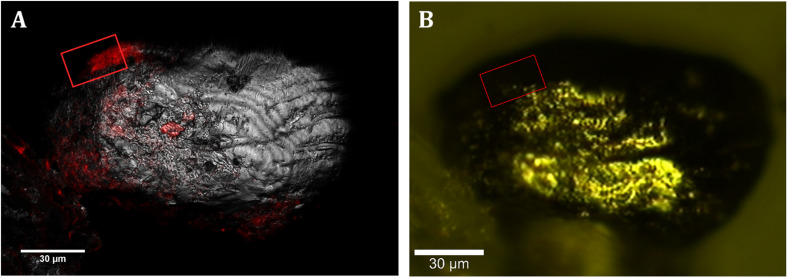
CLSM and CRM correlation. **(A)** Localization of members of *Acidovorax* spp. (red) attached to a mineral particle (in gray, reflection) at –228.6 m by CARD-FISH/CLSM. **(B)** Same mineral particle located in the CRM. Area analyzed by CRM is indicated by a red square. Scale bars, 30 μm.

To analyze the *Acidovorax*-minerals interaction, CARD-FISH experiments were repeated, but directly on the powdered rock samples, at those depths where the microorganisms were detected in the initial screening (Figure 2). Attached *Acidovorax* were only found at 6 of the 10 depths where these microorganisms were previously observed (Figure 1A). Once the *Acidovorax* colonies were located in the rock samples by fluorescence microscopy, the same area could be analyzed by CRM using the slide coordinate system (Figure 3). Raman analyses were carried out by mapping different focal planes (z-stack) of each studied area, in which a single spectrum/μm^2^ was acquired. As a result, for each analyzed area thousands of spectra were obtained that provided information on the structure and chemical composition of the sample in three dimensions with high spatial resolution.

**FIGURE 4:**
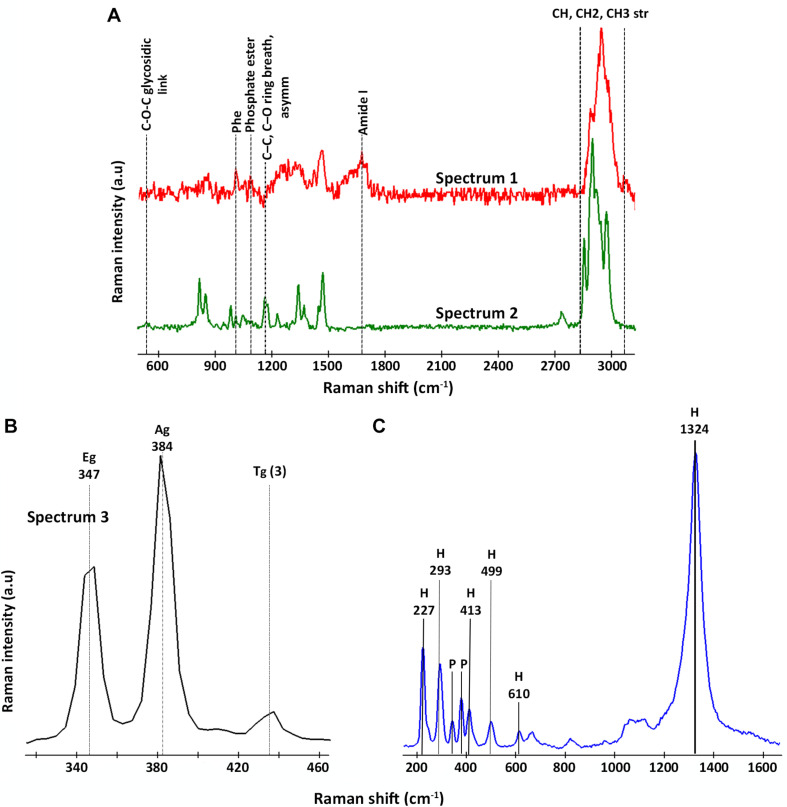
Representative Raman spectra detected by CRM in areas where members of the genus *Acidovorax* were located by CARD-FISH. **(A)** Raman spectra assigned to organic matter: in red, spectrum 1/*Acidovorax*; in green, spectrum 2/polysaccharides. **(B)** Raman spectrum assigned to pyrite (spectrum 3); and **(C)** Raman spectrum assigned to hematite. The characteristic bands of each spectrum are indicated.

**FIGURE 5:**
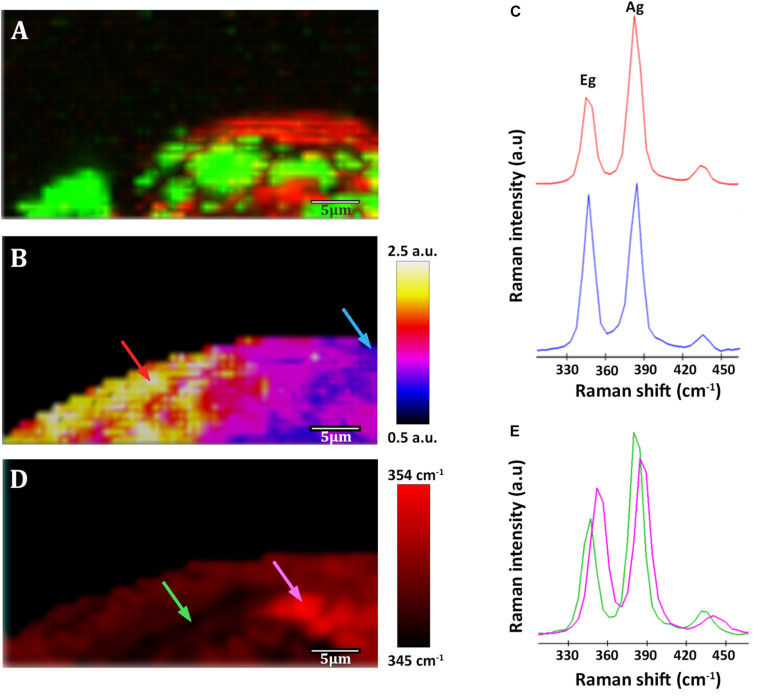
Raman images of a native sample from –228.6 m of the IPB subsurface (see Figure 3). **(A)** Raman map of organic matter. In red, distribution of *Acidovorax*. In green, distribution of polysaccharides. **(B)** Raman map colored according to the Ag/Eg ratio intensity of pyrite spectra. **(C)** Pyrite Raman spectra corresponding to the arrows marked with blue and red color in the image shown in panel **(B)**. **(D)** Map showing the Raman shift corresponding to the pyrite Eg band in a color code. **(E)** Pyrite Raman spectra corresponding to the arrows marked with green and pink in the image shown in panel **(D)**. Scale bar, 5 μm.

All the spectra could be classified into three representative groups, named spectra 1, 2, and 3 (Figures 4A,B). Both spectra 1 and 2 (Figure 4A) showed the characteristic bands of the CH stretching bond of organic matter in the range 2,800–3,020 cm^–1^ (Lin-Vien et al., 1991; Maquelin et al., 2002; Czamara et al., 2015). However, unlike spectrum 2, spectrum 1 presented Raman bands representative of different organic compounds such as proteins at 1,014 cm^–1^ and 1,672 cm^–1^, assigned to the presence of phenylalanine and to the amide I bond respectively (Ivleva et al., 2009; Rygula et al., 2013); and DNA and phospholipids at 1,092 cm^–1^, assigned to the phosphate ester bonds (Ivleva et al., 2009; Dieing et al., 2011). On the contrary, spectrum 2 is characterized by the presence of bands in the range of 530–540 cm^–1^ as well as 1,160 cm^–1^, which are representative of carbohydrates (Schuster et al., 2000; Ivleva et al., 2009; Wagner et al., 2009). Thus, we assigned spectrum 1 to the presence of microorganisms, in this case to members of the genus *Acidovorax* (previously identified by CARD-FISH) and spectrum 2 to polysaccharides. Figure 5A is a two-dimensional representation of the organic matter distribution in the analyzed area of a sample taken at 228.6 mbs, showing the presence of exopolysaccharides surrounding the cells.

On the other hand, spectrum 3 (Figure 4B) corresponds to the spectra of the mineral substrate to which the microorganisms were found attached. Spectrum 3 shows two bands of high intensity at 347 and 384 cm^–1^ and a less intense band at 435 cm^–1^, which are characteristic of the Raman-active modes of pyrite (Ushioda, 1972; Vogt et al., 1983; Bryant et al., 2018). The first corresponds to the S2 dumbbell libration (Eg), the second to the symmetric stretching of the S-S link in phase (Ag) and the third to the coupling of the libration and stretch modes [Tg (3)] (Blanchard et al., 2005). 95% of the examined *Acidovorax* colonies were found attached to pyrite. Of the 20 analyzed areas, only one exception to pyrite was observed at 139.4 mbs, in which an *Acidovorax* colony was attached to a mineral that could not be identified by its Raman spectrum (Supplementary Material 1).

In some cases, in addition to pyrite, traces of hematite were detected in the analyzed area of the sample. The hematite Raman spectrum (Figure 4C) is easily recognizable due to the presence of bands at 227, 246, 293, 412, 498, 610 cm^–1^ and, above all, the strong band at 1,322 cm^–1^, whose intensity varies with the intensity of the incident laser applied (De Faria et al., 1997; De Faria and Lopes, 2007).

### Pyrite Spectra Variability

**FIGURE 6:**
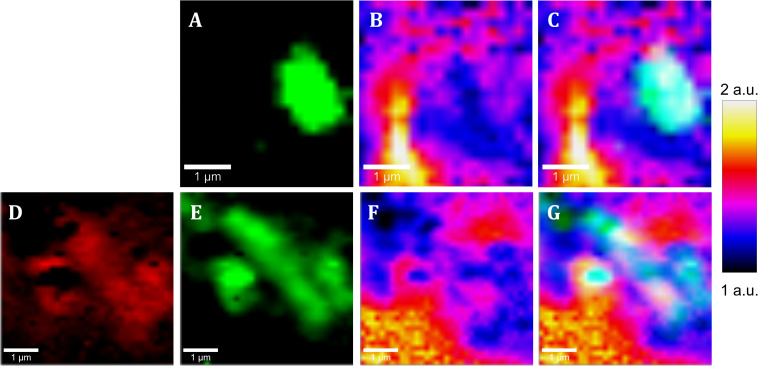
Raman images of native samples from the IPB subsurface at –139.4 m **(A–C)** and –206.6 m **(D–G)** showing the co-localization of cells and pyrite areas with low intensity ratio of Ag band relative to Eg band. **(A,E)** Raman map of *Acidovorax* (green). **(E)** Raman map of exopolysaccharides (red), **(B,E)** Raman map colored according to the Ag/Eg ratio intensity of pyrite spectra. **(C,G)** merge. Scale bar, 1 μm.

Pyrite Raman spectra vary from one sample to another and, interestingly, even within the same analyzed area its spectrum is not homogeneous. Variations at micrometer scale at the relative intensity of the Eg and Ag bands in the range of 0.8 ± 0.1–2.4 ± 0.2 (Figures 5B,C, 6B,E) were observed in individual pyrite grains, showing a mean value of 1.5 ± 0.2. In addition, changes in the pyrite bands positions up to 9 cm^–1^ (Figures 5D,E) were detected in the analyzed area of the pyrite grains, with mean values of 347.8 ± 0.8 and 383.9 ± 0.6 for Eg and Ag bands respectively.

To determine if there is any relationship between the occurrence of *Acidovorax* and the relative intensity of the pyrite Eg and Ag Raman bands or their position displacement, the variations of both parameters were analyzed in the presence or absence of the microorganism in each of the mappings performed.

**FIGURE 7:**
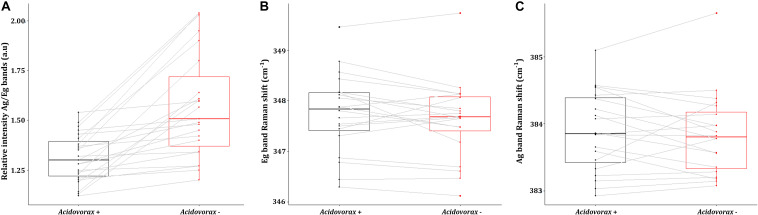
Average values of the intensity ratio of the Ag band relative to Eg band **(A)**, the Eg band Raman shift **(B)** and the Ag band Raman shift **(C)** of pyrite Raman spectra in presence (red) or absence (black) of *Acidovorax* resulting from Raman analysis of the IPB subsurface native samples.

As shown in Figures 6, 7A, it is clearly observed that there is an association between the location of *Acidovorax* and the low values of the relative intensities Ag/Eg of the pyrite spectra. In the presence of *Acidovorax*, the average of Ag/Eg ratio is 1.3 ± 0.1, while in the absence of *Acidovorax*, this value amounts to 1.6 ± 0.3. Statistics analysis corroborates the inverse correlation between *Acidovorax* Raman signal and the ratio Ag/Eg values of pyrite Raman spectra (PCC = −0.384 ± 0.111).

Regarding the Raman displacement of the Ag and Eg bands, the minimum difference in the average position of both bands in the presence and absence of the microorganism indicates that there is no correlation between the location of *Acidovorax* and this displacement (Figures 7B,C). Both in the presence and in the absence of *Acidovorax*, the positions of the Ag and Eg bands were 347.8 ± 0.8 and 383.9 ± 0.6 respectively.

### *Acidovorax* Biofilms Detection

**FIGURE 8:**
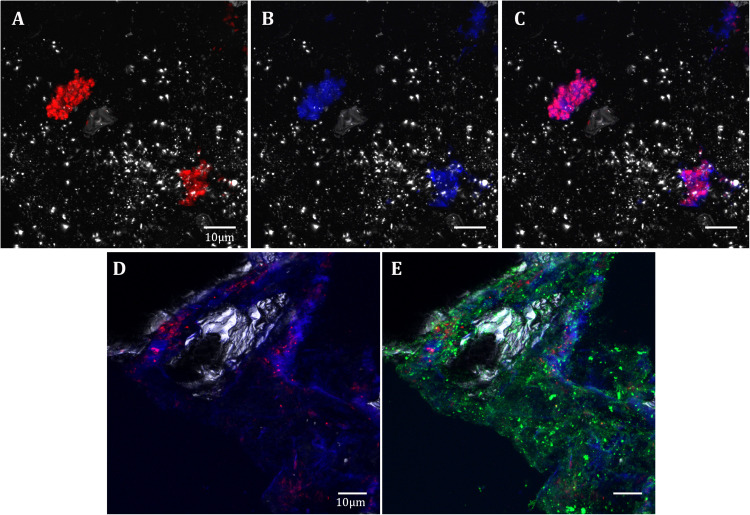
*Acidovorax*-Fe^3+^-EPS co-localization in the IPB subsurface. DOPE-FISH with *Acidovorax* probe (red) at 414.8 mbs **(A–C)** and at 139.4 mbs **(D,E)**; In blue, Fe^3+^ signal. In green, ConA lectin signal. In gray, reflection. Scale bars 10 μm.

Fluorescence Lectin Binding Assay (FLBA) was carried out to determine if *Acidovorax* colonies are included in biofilms as CRM suggest. FLBA confirmed that most *Acidovorax* colonies attached to the rock samples are surrounded by exopolysaccharides (Figure 8), which explain their detection by CRM in some analyzed areas (in Figures 5A, 6D). This observation corroborates the data presented in Escudero et al. (2018b), which indicated that biofilm formation is a common lifestyle of the rock attached microorganisms inhabiting the IPB subsurface. In addition, the use of a fluorescent iron sensor revealed that *Acidovorax* cells are surrounded by ferric iron (Figure 8).

## Discussion

Both the distribution and the microorganism-mineral relationship of *Acidovorax* genus in the IPB subsurface, a geological formation considered one of the largest massive sulfide deposits in the world (Tornos, 2006), have been analyzed in this work. *Acidovorax* was chosen for being one of the most abundant iron oxidizer microorganisms detected in the IPBSL (Amils et al., under review), a drilling project devoted to the study of the underground bioreactor responsible for the peculiarities of Río Tinto (Amils et al., 2013). Our CARD-FISH results indicated that, indeed, *Acidovorax* is a genus with a high distribution along the BH10 column (Figure 1A). This data together with the observation of a high distribution of *Acidovorax* in the MARTE project (Puente-Sánchez et al., 2014), performed also in the IPB subsurface, strongly suggest that this genus must play an important role in the iron and nitrogen cycles of this ecosystem.

To determine if there is any relation between the distribution of *Acidovorax* and the IPB subsurface mineralogy throughout BH10, we used Raman-FISH technique. Fluorescence microscopy allows a specific microorganism to be identified by using specific probes (Amann et al., 1995) while confocal Raman microscopy analyses the composition and molecular structure of the mineral substrate (Smith and Dent, 2013). Thus, while FISH provided the specific location of *Acidovorax* genus members, the mineral to which it is attached was identified by means of Raman spectroscopy. To perform Raman-FISH assay, CARD-FISH was carried out directly on the powdered rock samples (Figures 2, 3). However, unlike the CARD-FISH performed on filter membranes, *Acidovorax* was only observed at 6 depths. This decrease may be due either to the heterogeneity of the sample, thus we could never analyze exactly the same sample even if it came from the same depth; or to the different protocol that has been applied to the sample depending on whether CARD-FISH is carried out on the membrane or directly on the solid substrate. In the first case, the microorganisms observed are those that have been detached from the rock by sonication while in the second case the microorganisms detected are those that remain attached after rock grinding. In addition, because the agarose interference is to be avoided in the Raman analyses, the rock samples were not immobilized. Thus, the microorganisms could have been detached from the rock surface during the different washes and treatments carried out throughout the CARD-FISH protocol. Consequently, the number of microorganisms remaining attached could be lower in agarose-free than in immobilized rocks, as well as the probability of detecting *Acidovorax* in a given depth when Raman-FISH protocol was applied.

All the detected *Acidovorax* colonies, but one at 139, 4 mbs, were attached to pyrite in each of the analyzed samples despite the array of different minerals detected along the column (Figure 1A and Supplementary Material 2). This data strongly suggests that the distribution of members of this genus in the system is related to the presence of this iron sulfide along the column. Members of the genus *Acidovorax* are nitrate reducers which are able to oxidize iron. These microorganisms were originally described as mixotrophs, capable of using iron as electron donor and acetate as carbon source (Straub et al., 2004; Kappler et al., 2005). However, the presence of iron is not essential for microorganism growth (Muehe et al., 2009; Chakraborty et al., 2011) and no proteins related with iron oxidation were detected in *Acidovorax* (Carlson et al., 2013). Thus, it was proposed that Fe^2+^ is most likely indirectly oxidized by these microorganisms through the reactive nitrogen species produced during the denitrification process (Picardal, 2012; Klueglein and Kappler, 2013; Klueglein et al., 2015). Even so, enzymatic oxidation of iron has not been ruled out and both processes, biotic and abiotic, are accepted today (Carlson et al., 2013; Schaedler et al., 2018). *Acidovorax* colonies in the IPB subsurface can be found surrounded by ferric iron (Figure 8), indicating that it was oxidized by the microorganism. Due to the ability of *Acidovorax* to indirectly oxidize iron, the possibility that the ferric iron produced could oxidize pyrite (Vera et al., 2013) even at neutral pH (Moses et al., 1987; Moses and Herman, 1991) cannot be discarded. Actually, detected Fe^3+^ by fluorescence microscopy was mostly co-localized with exopolysaccharides (Figure 8B), which are commonly responsible for metal ions binding and are essential to enhance the biooxidation of metallic sulfides (Sand and Gehrke, 2006). Such a reaction could result in the dissolution of the mineral and the liberation of sulfur compounds and ferrous iron, which may be seized by the microorganism as an electron donor, a nutrient or a detoxifying agent as suggested by Klueglein and Kappler (2013) and Carlson et al. (2013) respectively. Indeed, it has been shown that *Acidovorax* growth is improved by the presence of ferrous iron (Chakraborty et al., 2011). Therefore, the attachment of *Acidovorax* to pyrite and its subsequent dissolution could be an advantage for the microorganism in this oligotrophic environment. The detection of hematite, a ferric iron oxide whose appearance was observed after the dissolution of pyrite as a secondary mineral (Caldeira et al., 2003), in some of the analyzed samples, would support this hypothesis.

Pyrite dissolution by denitrifying microorganisms has been of interest for the past few decades due to its environmental interest (Postma et al., 1991; Schwientek et al., 2008; Zhang et al., 2009) and several studies support that pyrite can be used as electron donor by denitrifying chemolithotropic microorganisms (Jørgensen et al., 2009; Torrentó et al., 2010, 2011; Bosch et al., 2012; Vaclavkova et al., 2015). Nevertheless, preliminary studies showed that *Acidovorax* BoFeN1 is not capable of dissolving pyrite or, at least, does not produce sulfate increment into the medium when it grows in the presence of this mineral (Yan et al., 2019). It should be noted, however, that other sulfur species such as thiosulfate or tetrathionate, which are more stable at neutral pH, may have been released into the medium instead of sulfate (Moses et al., 1987; Moses and Herman, 1991). Thus, more in depth analysis would be needed to unequivocally demonstrate whether *Acidovorax* is able to biooxidize pyrite.

In our Raman analysis, variations related with the displacement and the relative intensity of the Eg and Ag bands in the Raman spectrum of native subsurface pyrite were observed at micrometer scale (Figures 5B–E, 6B,E). Actually, despite the absence of relation between the location of *Acidovorax* and the displacement of Raman pyrite bands (Figures 7B,C), our data indicate that there is a correlation between the location of *Acidovorax* and the low values of the relative intensity Ag/Eg of the pyrite spectra (Figures 6, 7A).

Unfortunately, as far as we know, there is no data available about spatially resolved surface characterization of pyrite by CRM with which to contrast our results. All measurements made up to now were carried out with macroscopic techniques which provide only averaged pyrite spectra of a relatively large area (Bryant et al., 2018 and references therein). Still, the causes that may explain the differences in the pyrite Raman spectra observed between different studies has been analyzed in depth by Bryant et al. (2018). On the one hand, the displacement of the position of the pyrite bands toward lower wavelengths has been attributed either to an effect of laser heating or to the presence of trace elements such as copper, zinc or lead among others (Bryant et al., 2018). While in the latter case the variation of the position of the bands is minimal (up to ∼ 1 cm^–1^), downshifting of the pyrite bands up to 12.7 cm^–1^ due to the laser heating has been reported (Bryant et al., 2018). However, these extreme variations in bands position have only been detected by modifying the laser power in the analysis of pyrite of a grain size below 10 μm. In our analysis, in which a constant low laser power was used, variations up to 9 cm^–1^ in the position of the pyrite were detected (Figures 5D,E). Hence, the observed Raman shifts in the pyrite grains analyzed in this work cannot be attributed to laser heating and further analysis are necessary to understand this phenomenon.

On the other hand, changes in the relative intensity of the Eg and Ag bands have been attributed to the crystalline orientation of pyrite with respect to the polarization plane of the incident laser, which, in addition, may be affected by the laser power. Bryant and collaborators showed variations in the range of 0.22–1.3 in centimeter scale cubic pyrite while in pyritohedral pyrite (12 faces) they varied from 0.81 to 2.01 due to the differential excitation of the bands of the diverse analyzed pyrite faces. In fact, the mean value of the intensity ratio Eg/Ag band varies from 0.9 to 3.5 in studies in which pyrite of varied sizes and morphologies were analyzed using a range of laser powers (Bryant et al., 2018 and references therein). Accordingly, the microscale variations of the intensity ratio Ag/Eg observed in our study (Figures 5B,C, 6B,E) could be due to the fact that the analyzed pyrite particles present a polycrystalline structure. Thus, different faces could have been mapped in the same Raman analysis. Accordingly, the low value Eg/Ag preference by the microorganism could indicate that *Acidovorax* is attached preferentially to those faces of pyrite in which the band Eg, which represents the S2 dumbbell vibration of the pyrite structure, shows a greater intensity (Figure 6). However, the possibility that *Acidovorax*, in some way, could affect the pyrite structure should not be ruled out and we would like to clarify this interesting point in the future.

## Conclusion

Since rocks are the matrix in which microorganisms live in subsurface, it is feasible to assume that their composition might influence the microbial distribution in this environment. Our Raman-FISH results suggest a strong relationship between the distribution of *Acidovorax* and pyrite, which may be explained by the microorganism’s metabolic preference for pyrite. In addition, heterogeneity in uncolonized vs. *Acidovorax*-associated pyrite Raman spectra was observed. One possible explanation for this observation is the modification of the pyrite by *Acidovorax*. However, the lack of information about a possible biological alteration in pyrite Raman spectrum at the micrometer scale makes the interpretation of these results difficult. Thus additional experiments are needed to analyze any advantages to *Acidovorax* growing in the presence of pyrite or dissolution of the mineral carried out by the microorganism with the possible fingerprints resulting from their interaction. Nevertheless, correlative fluorescence and confocal Raman microscopy has been shown to be a useful method with which to analyze, *in situ*, the mineral dependent distribution of the subsurface biodiversity.

## Data Availability Statement

The raw data supporting the conclusions of this article will be made available by the authors, without undue reservation.

## Author Contributions

CE wrote the manuscript. CE and RA designed the study and interpreted the results. CE and JM carried out the fluorescence *in situ* hybridizations and fluorescence microscopy observations. AC performed confocal Raman microscopy analyses. CE, AC, CS, JA, and RA interpreted the Raman part of the study. RA, AC, CS, JA, JM, and FG corrected the manuscript. All authors contributed to the article and approved the submitted version.

## Conflict of Interest

The authors declare that the research was conducted in the absence of any commercial or financial relationships that could be construed as a potential conflict of interest.
