# Supplementary figures and images for: Visualizing Microorganism-Mineral Interaction in the Iberian Pyrite Belt Subsurface: The Acidovorax Case
Source: Front Microbiol. 2020 Nov 26;11:572104. doi: 10.3389/fmicb.2020.572104 (PMC7726209; doi:10.3389/fmicb.2020.572104)

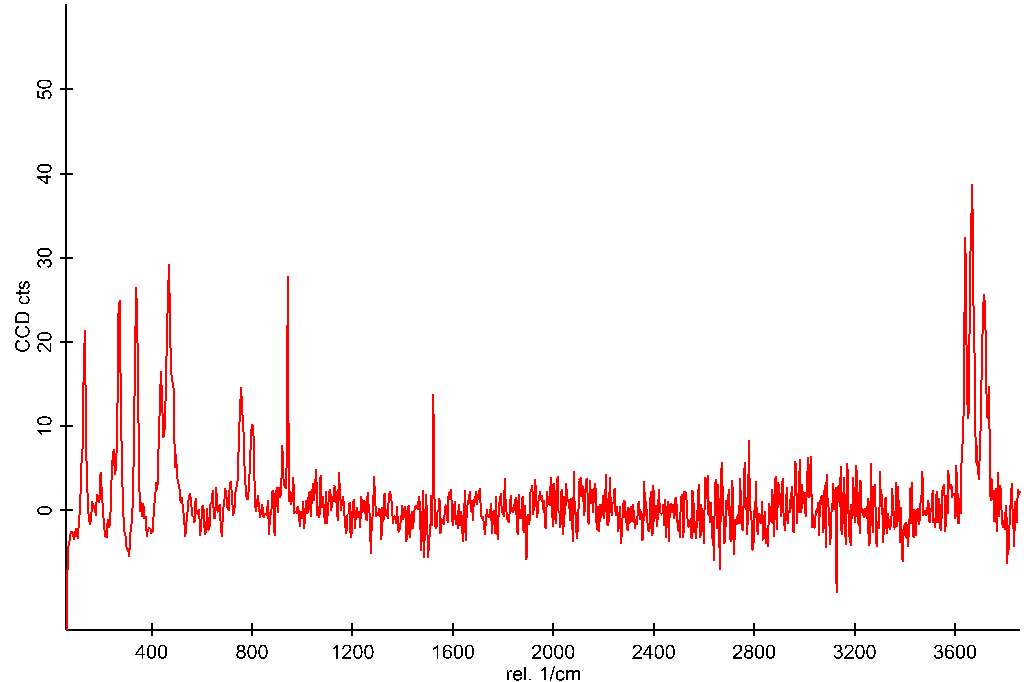

Supplement: Supplementary file 2 [file Image_1.TIFF]
